# Rationale for Recommending Varicella Vaccination Into the Swedish National Immunisation Programme

**DOI:** 10.1111/apa.70497

**Published:** 2026-03-26

**Authors:** Annika Ersson, Kari Johansen, Sören Andersson

**Affiliations:** ^1^ Department of Public Health Analysis and Data Management Public Health Agency of Sweden Solna Sweden; ^2^ Department of Communicable Disease Control and Prevention Region Jämtland‐ Härjedalen Östersund Sweden

**Keywords:** age, catch‐up vaccination, equitable access, legal prerequisites, varicella vaccination

## Abstract

**Aim:**

Under Swedish law, a vaccination may be included in the National Immunisation Programme (NIP) if it effectively prevents transmission in the population, is cost‐effective and aligns with ethical and humanitarian principles. The Public Health Agency of Sweden (PHAS) evaluated these criteria for introducing varicella vaccination.

**Methods:**

The evaluation included a literature review, disease modelling to assess dose timing, a health economic analysis and an ethical review addressing vaccine acceptance and equitable access.

**Results:**

Varicella vaccines are safe and highly effective, providing near‐complete protection against severe disease requiring hospitalisation when two doses are given. Modelling indicates that virus circulation could be eliminated within 2–3 years with high coverage and catch‐up vaccination, also protecting unvaccinated and immunocompromised individuals of all ages through population immunity. Two doses are recommended at 18 months and 7–8 years, both co‐administered with MMR. This schedule ensures durable immunity and high acceptance. Health economic analyses show the programme would be cost saving.

**Conclusion:**

Following PHAS's evaluation, the Swedish government has decided to include varicella vaccination in the NIP from 2027, with catch‐up vaccination for susceptible individuals up to 18 years, implemented primarily through the child and school health services to ensure equitable access.

## Summary

- A review of the prerequisites for including varicella vaccination in the Swedish National Immunisation Programme (NIP) was performed.
- The review showed that the programme is likely to reduce the spread of varicella in a few years' time and is health economically favourable, and with catch‐up vaccinations, the risk of rebound infections can be avoided.
- Varicella vaccinations given at 18 months and 7–8 years will be part of Sweden's NIP from 2027.

## 1 Introduction

The first varicella vaccines were launched in the late 1980s and incorporated into national immunisation programmes (NIPs) starting in 1995 in the United States [1], followed by Uruguay and Canada in 1999 [2, 3]. Since then, more than 45 countries worldwide have included varicella vaccination in their NIPs, including around a dozen in the European Union. In Europe, Germany was the first country to add varicella vaccination to its NIP in 2004 [4].

In the Nordic countries, Finland incorporated varicella vaccination into its NIP in 2017 at 18 months and 6 years of age [5], followed by Iceland 3 years later, which currently provides vaccination at 18 months and 2.5 years of age [6]. Finland has reported vaccination coverage rates of above 85% for the first dose and close to 60% for the second [5]. In Iceland, coverage rates above 90% have been achieved for both doses (K Sigriður Jósefsdóttir, Directorate of Health, Iceland, personal communication).

The Public Health Agency of Sweden (PHAS) is responsible for the implementation and follow‐up of the regulations concerning NIPs, while the actual delivery is carried out by the regional authorities in accordance with recommendations and legal requirements issued by the PHAS. Based on a recent evaluation, the PHAS has concluded that the prerequisites for including varicella vaccination in the Swedish NIP are met [7]. The evaluation included assessment of burden of disease, modelling of the potential impact of introducing varicella vaccination in the NIP in Sweden, as well as health economic analyses.

## 2 The Legal Prerequisites

Swedish NIPs are regulated under the Communicable Diseases Act (SFS 2004:168 and SFS 2015:159, 7§), which stipulates that a communicable disease may be included in a NIP if vaccination against the disease is expected to:

- Effectively prevent the transmission of communicable diseases within the population.
- Demonstrate cost‐effectiveness from a societal and health economic perspective and
- Be sustainable when assessed against ethical and humanitarian principles.

The PHAS is responsible for conducting evaluations and presenting recommendations to the government regarding the introduction of vaccinations against new infectious diseases. The government then makes the final decisions. In assessing whether the relevant conditions are met, the vaccine under consideration is evaluated against the following 13 criteria:

- The burden of disease in society, within the health care system, and for individual patients
- The expected impact of vaccination on the disease burden and on the epidemiology of the disease
- The number of doses required to achieve the intended effect,
- The target groups to be offered vaccination,
- The safety profile of the vaccine,
- The impact of vaccination on the operations of regional, municipal and private health care providers,
- The suitability of the vaccine for integration with other vaccines in the national immunisation programmes,
- The public's acceptance of the vaccine and its potential influence on attitudes towards vaccination more broadly,
- Other available preventive measures or treatments that may be implemented or offered as alternatives to vaccination within a national immunisation programme,
- The socio‐economic effects of vaccination, including its costs and benefits for the state, municipalities and county councils,
- The possibilities for monitoring the effects of vaccination with respect to the factors outlined in points 1–10, as well as the estimated costs for conducting such monitoring,
- The need for public and provider information campaigns and the associated costs of these measures,
- Medical‐ethical and humanitarian considerations.

## 3 Disease Burden of Varicella Zoster Virus Infections

In non‐tropical countries without varicella vaccinations in NIPs, most children become infected with varicella zoster virus (VZV) during early childhood. In Sweden, the peak age of infection is estimated to be during day care age [8, 9]. By 12 years of age, more than 90% of children would have acquired immunity [9, 10]. These findings are consistent with statistics on parental leave benefits from the Swedish Social Insurance Agency (Försäkringskassan) [11].

Varicella is generally considered a fairly mild disease, although severe complications, such as encephalitis and cerebellitis, may occur [9]. According to Widgren et al., dehydration, bacterial infections and septicemia as well as neurological involvement were the most frequent complications in children with more severe varicella infection [9].

Swedish studies using national and regional data from 2007 to 2013 reported a hospitalisation rate of 20–41 per 100 000 person years (PY) among children up to 5 years of age, while the consultation rate in primary care was 109 per 100 000 PY, with most cases occurring in 2‐year‐olds [8]. The average duration of hospital stay was 3–5 days according to data from the Swedish National Board of Health and Welfare and increased with age. Mortality due to varicella, or with varicella as a contributing factor, is low, although particularly vulnerable individuals, that is, with pre‐existing medical conditions of immunodeficiency, may develop life‐threatening conditions [12].

After primary infection, VZV remains dormant in the nervous system throughout life. It is estimated that the virus reactivates to cause herpes zoster (shingles) with a lifetime risk of approximately 30%–40% of individuals previously infected (risk increasing with age) [13]. Recent studies also indicate that VZV may play a role in the development of dementia, and thus, zoster vaccines may reduce or delay dementia [14, 15, 16], although there is consensus that further research is required before such an association can be considered in models of public health impact and health economic analyses.

## 4 Varicella Vaccines Available

Four varicella‐containing vaccines are approved either in Sweden or in the EU. They are manufactured by two vaccine producers, each of whom markets one monovalent varicella (V) vaccine and one tetravalent measles‐mumps‐rubella‐varicella (MMRV) vaccine. All of these four vaccines contain live attenuated VZV.

The available vaccines against varicella are safe and effective, providing near‐complete protection against varicella disease requiring hospitalisation when two doses are administered. In addition, circulation of the virus in the community is substantially reduced if vaccination coverage is high [5]. Varicella vaccines have been in use for several decades, and the longest follow‐up to date has shown that immunity lasts for at least 10 years, largely irrespective of age at vaccination or vaccine type. A meta‐analysis of outbreak data reported an overall vaccine effectiveness (VE) of 72.5% after a single dose [17]. Some waning of immunity can be expected between the first and second doses; the impact of which will depend on the extent of virus circulation in the population. Limited varicella outbreaks have been reported following the implementation of one‐dose schedules [18]. Further studies on long‐term effects of varicella vaccinations are needed, beyond the current ten‐year limit.

## 5 Time Points of Two Vaccine Doses and Catch‐Up Vaccination

In the first national varicella vaccination programmes introduced in countries such as the United States, Germany and Canada, a single vaccine dose was offered, leading to significant reductions in morbidity and mortality. However, as regular outbreaks continued to occur even among vaccinated children, a second dose was subsequently added [19, 20]. One dose has therefore been shown to be sufficient to markedly reduce morbidity and mortality, but two doses are required to prevent continued virus circulation and breakthrough varicella infections, which is the goal for the introduction of a vaccination programme in Sweden.

The first dose is usually administered between 12 and 18 months of age, with the interval to the second dose varying but most commonly between 18 months and 6 years of age [21]. The timing and spacing of the doses depend on several factors, including the scheduling of other vaccines (for administration logistics), the epidemiology of varicella in the country, waning immunity after the first dose (to avoid breakthrough infections in the interval before the second dose) and expected vaccine acceptance among caregivers.

The waning immunity after a single vaccine dose is one reason why some countries have chosen to offer both doses at a short interval, in order to avoid outbreaks caused by breakthrough infections while awaiting the second dose. However, the risk of breakthrough infections can be substantially reduced by offering catch‐up vaccinations to all children and adolescents who remain susceptible [5, 22] while keeping a longer interval between doses. As the number of susceptible individuals decreases, virus circulation in the community will be diminished. According to PHAS modelling, virus circulation could be eliminated in just 2–3 years, provided that catch‐up vaccination is offered and vaccination coverage is sufficiently high. The risk of outbreaks is then expected to be low due to very limited virus circulation, making it possible to extend the interval between doses, for example, aligning with both MMR doses without risking a resurgence of varicella infections.

The Swedish modelling results are consistent with the experience in Finland, which introduced universal varicella vaccination in 2017 with two doses at 15 months and 6 years of age, along with a complementary catch‐up vaccination offered to all susceptible children up to 12 years of age [5]. Compared with the years preceding universal varicella vaccination (2014–2016), the 2022 consultation rate in the primary health care for varicella‐related causes in Finland was reduced across all age groups—by 92%–98% among children eligible for vaccination and by 87% among unvaccinated children under 12 months of age [5]. This outcome illustrates how susceptible individuals are indirectly protected through reduced virus circulation following introduction of a national immunisation programme against varicella.

In the Swedish context, vaccinations at a child health centre are scheduled at 6 weeks, 3, 5, 12 and 18 months, and at 5 years of age. There are also scheduled visits at 2.5–3 and 4 years of age, during which no vaccinations are planned. Thereafter, children are transferred to the school health services, where vaccinations are provided at 7, 10 and 15–16 years of age (Table 1).

**TABLE 1:** The table shows when vaccinations in the Swedish national immunisation programme are offered within child and school health services before varicella vaccination is added.

Vaccine against	6 weeks	3 months	5 months	12 months	18 months	5 years	School year 1–2	School year 5	School year 8–9
Rotavirus	Dose 1	Dose 2	Dose 3	—	—	—	—	—	—
Diphtheria^a^	—	Dose 1	Dose 2	Dose 3	—	Dose 4	—	—	Dose 5
Tetanus	—	Dose 1	Dose 2	Dose 3	—	Dose 4	—	—	Dose 5
Pertussis	—	Dose 1	Dose 2	Dose 3	—	Dose 4	—	—	Dose 5
Polio	—	Dose 1	Dose 2	Dose 3	—	Dose 4	—	—	—
*Haemophilus influenzae* type b	—	Dose 1	Dose 2	Dose 3	—	—	—	—	—
Pneumococci	—	Dose 1	Dose 2	Dose 3	—	—	—	—	—
Measles, mumps, rubella	—	—	—	—	Dose 1	—	Dose 2	—	—
Human papillomavirus (HPV)								Dose 1 and 2	

^a^ In addition, all Swedish children are offered hepatitis B vaccination as part of the hexavalent vaccine containing diphtheria, tetanus, pertussis, polio, hepatitis B, hemophilus influenzae used in all regions, despite no formal national recommendation being available.

Several ages were considered suitable for offering the two doses of varicella vaccine in Sweden. One option was to give the two varicella vaccine doses at 12 and 18 months of age, with the first dose administered before most children enter day care and the second dose offered soon thereafter. This approach would also avoid waning immunity during the short interval between the two doses. On the downside, however, the child would receive three separate injections at the 12‐month visit, which could negatively affect vaccine acceptance.

If the first dose is given at 18 months of age, there are several possible timings for the second dose. The visits at 3 or 4 years of age were deemed unsuitable, as no vaccinations are currently offered at these ages. The 5‐year visit would be a feasible option, as would the school appointment for the second MMR dose. Despite the higher risk of breakthrough varicella due to the longer interval between doses, it was considered preferable to align the second dose with the MMR vaccine, given the possibility of combining them in a single injection using the combination vaccine MMRV. The potential risk of breakthrough varicella infection with a longer interval between doses one and two can be mitigated through a catch‐up programme, as further described in this article.

Health care professionals in Sweden also emphasize the importance of not administering vaccinations at every child health care visit, in order to avoid children associating such visits with painful injections. These considerations are important when planning the optimal timing of the two vaccine doses in a varicella vaccination programme. The number of injections can be reduced if varicella vaccination is coordinated with measles, mumps and rubella (MMR), by offering a tetravalent MMRV vaccine instead of adding a monovalent varicella (V).

Health economic analysis showed that implementing any of the evaluated schedules would be favourable, that is, cost saving from a societal perspective, while from a health system perspective, a cost per gained Quality‐Adjusted Life Year (QALY) of around Euro 20 000 is estimated (article in this supplement).

## 6 Monovalent (V) or Tetravalent (MMRV) Vaccine?

From an immunological perspective, administering several vaccines at the same time is usually not problematic. However, both guardians and health care staff may have concerns regarding the number of vaccines given to a child during a single visit to a child health centre, not least due to the discomfort for the vaccinee from receiving many injections at once.

The tetravalent MMRV vaccines confer similar vaccine effectiveness as the monovalent vaccines, but have been associated with an increased risk of febrile convulsions when administered as the first dose in young children. Febrile convulsions are usually benign and may also occur with infections, such as varicella. The risk has been estimated at approximately 1 case per 2500 vaccinations among children 12–23 months of age, which is about twice as high as when using the monovalent varicella vaccine for the first dose [23]. Numerically, the increased risk is small and may not warrant exclusion of MMRV as the first‐dose option. Many countries, including the United States and Canada, use the MMRV vaccine for both the first and second doses. In November 2023, the British Joint Committee on Vaccination and Immunisation (JCVI) proposed introducing varicella vaccination with MMRV in the childhood vaccination programme, and it will now be initiated in January 2026 [24]. The risk of febrile convulsions is not increased after the second MMRV dose.

If much attention is given, for example, in social media, traditional media and other channels to the small, but nevertheless increased risk of febrile convulsions when MMRV is administered as the first dose, it may raise questions among some guardians. It is also important that guardians retain the option to accept only the MMR vaccine offer if they are reluctant to consent to varicella vaccination. If MMRV was the only option, there would be a risk that reluctant guardians might choose not to vaccinate at all, which could negatively affect MMR coverage. There is a generally high confidence in the NIP in the Swedish population with stable coverage rates above 90%. A study on guardians' and health staff's acceptance of the addition of varicella vaccination to the Swedish NIP is presented in the modelling article (article in this supplement). In brief, it showed that a majority of the parents and health staff were positive to including varicella vaccinations in the NIP, regardless of the age of the children.

## 7 Vaccination Provides Indirect Protection to Susceptible Individuals

The VZV is expected to stop circulating in society a few years after a vaccination program is introduced. The exact number of years will depend on how many children and adolescents remain susceptible in the respective age cohort. When susceptible children and adolescents of all ages are vaccinated, virus circulation in society will cease within a few years due to the high level of population immunity, provided that a high vaccination coverage is reached in a relatively short time.

Varicella disease is often milder in young children than in teenagers and adults. A successful vaccination programme that reduces varicella virus circulation may therefore have a negative impact on those children who were not vaccinated and have not yet contracted varicella, as their risk of reaching adulthood without immunity will increase. On the other hand, a small percentage of adults remain susceptible to varicella virus even without a vaccination programme. For these individuals, reduced virus circulation would be indirectly protective, as their risk of contracting varicella decreases accordingly.

For immunocompromised individuals of all ages, the reduced circulation of the varicella virus following the introduction of a vaccination programme will also provide indirect protection and be of benefit until many of them regain their immunity through vaccination [12].

## 8 Organising Catch‐Up Vaccination

While routine childhood vaccinations in Sweden are commonly administered through a specialised child or school health organisation, a temporary catch‐up vaccination programme may need to be organised separately. One approach could be to offer two doses of the vaccine to all susceptible children up to a certain age within a limited time period following the start of the vaccination programme. Another approach may be to offer the varicella vaccine at all ordinary vaccination visits until the catch‐up programme is complete.

Individuals who have had varicella or have previously received two vaccine doses are considered immune to the varicella virus, provided the infection or vaccination did not occur before 12 months of age, since the infant immune system may not be mature enough to develop long‐lasting immunity. As the varicella vaccine can be considered safe to administer regardless of if the recipient has been exposed to the virus before or not, there is no need to verify varicella immunity status prior to vaccination. Caregiver recall will thus be sufficient to assess whether a child is immune or susceptible to the VZV. An option could be to offer the vaccine to all children regardless of prior vaccination or infection, which may simplify the organisation by eliminating the need to collect vaccination history from caregivers.

## 9 Ethical Aspects of Equal Vaccination Offers

Ethical aspects of varicella vaccination were reviewed by the Swedish National Council on Medical Ethics [25]. Vaccines included in the Swedish NIP are offered through child and school health services at no extra cost to families. Vaccine coverage in Sweden has been high and stable for many years, with well over 90% for at least the first dose of all included vaccines. For MMR, diphtheria, tetanus, pertussis and pneumococcal vaccines, this high coverage also extends to subsequent doses. School‐based vaccination programmes help ensure equal access to vaccines, as acceptance is less likely to be influenced by parents' financial situation or their ability to bring the child to a vaccination centre.

In the Swedish context, organising a catch‐up vaccination is likely to be more equitable if the vaccine doses are offered through child and school health services, rather than for example, by providing the vaccine only at certain vaccination centres. The latter approach will rely on guardians taking the initiative, which could lead to disparities influenced by economic status, education level and how well information is communicated, ultimately affecting vaccination coverage.

## 10 Proposal From the Public Health Agency of Sweden and the Subsequent Government Decision

In conclusion, based on the considerations above, the PHAS has proposed to include two doses of varicella vaccine in the Swedish childhood vaccination programme, administered at the following ages:

- Dose 1 at 18 months, together with MMR.
- Dose 2 at 7–8 years, together with MMR.

Should the second MMR vaccine dose in the future be moved to an earlier age, such as 6 years of age, it is proposed that the varicella‐containing vaccine be moved at the same time.

As the varicella vaccine is proposed to be offered at the same age as MMR, it is possible to use the tetravalent MMRV vaccine. However, the PHAS does not recommend any of the available vaccine products over the others, as all four options are considered equal in terms of safety and effectiveness.

In September 2025, the Swedish government decided to include varicella vaccination in the Swedish National Immunisation Programme starting in January 2027. This includes support for catch‐up vaccination of all susceptible children and adolescents up to 18 years of age who are not eligible for vaccination within the routine programme, that is, those who have passed the age for receiving the two scheduled doses. This is intended both to quickly eliminate virus circulation in society and to reduce the risk of susceptible children and teenagers reaching adulthood without immunity to varicella.

## Author Contributions

**Annika Ersson:** conceptualization, methodology, project administration (lead), writing – original draft (lead). **Kari Johansen:** conceptualization (supporting), supervision (supporting), validation, writing – review and editing (supporting). **Sören Andersson:** conceptualization, writing – review and editing (lead), methodology, project administration (supporting), supervision (lead), resources, validation.

## Data Availability

Data sharing not applicable to this article as no datasets were generated or analysed during the current study.
